# Rapid Detection of microRNA-122 in Serum and Finger Blood Using a Lateral Flow Nucleic Acid Biosensor

**DOI:** 10.3390/bios15010058

**Published:** 2025-01-17

**Authors:** Min Zhang, Meijing Ma, Jiahui Wang, Yurui Zhou, Xueji Zhang, Guodong Liu

**Affiliations:** 1 School of Chemistry and Chemical Engineering, Linyi University, Linyi 276005, China; 2 Marshall Laboratory of Biomedical Engineering, Research Center for Biosensor and Nanotheranostic, School of Biomedical Engineering, Shenzhen University, Shenzhen 518060, China

**Keywords:** miR-122, lateral flow, biosensors, gold nanoparticles, visual detection

## Abstract

MicroRNA122 (miR-122) is a microRNA that is highly expressed in hepatocytes and has been identified as a prospective therapeutic target and biomarker for liver injury. An expanding body of research has demonstrated that miR-122 is a critical regulator in both the initiation and progression of a wide range of liver diseases. Traditional methods for detecting miR-122 mainly include Northern blotting and qRT-PCR, but they are technically complex and cumbersome, requiring expensive instruments and high technical requirements. In this paper, we present a novel rapid testing method utilizing a lateral flow nucleic acid biosensor (LFNAB) for the sensitive and time-efficient detection of miR-122. This approach offers several advantages, including a high specificity for miR-122, the ability to detect low concentrations of the target molecule, and a significantly reduced testing time compared to conventional detection methods. In this study, a thiol-modified single-stranded detection DNA probe (Det-DNA), a biotinylated single-stranded capture DNA probe (Cap-DNA), and a biotinylated single-stranded control DNA probe (Con-DNA) are used to construct the LFNAB. A gold nanoparticle (AuNP) is a colored tag, which is used to label the Det-DNA probe. The principle of detecting miR-122 is based on dual DNA-miRNA hybridization reactions on the LFNAB to form sandwich-type AuNP-Det-DNA-miR-122-Cap-DNA complexes, which are captured on the test area of LFNAB for visualization and quantification. After systematic optimization of conditions of experiment, the response of LFNAB was highly linear within the scope of 0 pM-100 pM miR-122, and the detection limit in 15 min was 3.90 pM. The use of LFNAB to detect miR-122 in serum and fingertip blood has yielded satisfactory results. This successful application indicates the effectiveness of LFNAB in detecting miR-122 in both serum and fingertip blood samples, showcasing its potential utility in clinical and research settings for assessing miR-122 levels in different biological samples.

## 1. Introduction

MicroRNAs (miRNAs) are a kind of small non-coding RNA molecule, typically around 22 nucleotides in length, and known to modulate gene expression at the post-transcriptional level across a wide array of cell types [1]. Altered miRNA expression has been linked to specific liver conditions, including hepatitis, hepatocellular carcinoma, and cirrhosis [2]. MiRNA-122 exhibits high expression levels in the liver, representing approximately 70% of the total miRNA pool [3]. A growing body of research shows that miRNA-122 plays a significant role in the initiation and advancement of various liver diseases. Therefore, miRNA-122 can be used as a biomarker for liver injury. Traditional miRNA-122 detection methods include Northern blotting, in situ hybridization, RT-qPCR, microarray, and next-generation sequencing, etc. [4]. Northern blotting demands a substantial amount of test samples and utilizes radioactive probes, making it unsuitable for the detection of low-abundance samples [5]. In situ hybridization requires complex steps such as denaturation, long reaction times, and specific software or fluorescence imaging microscopy to detect the probe itself. RT-qPCR offers a high sensitivity; however, the process of sample preparation is complex and, more importantly, constrained by the short length of miRNA sequences [6]. Expression profiling based on microarrays offers only a semi-quantitative evaluation of gene expression; it is constrained by the types of probes included in the platform and the limitations of their sensitivity and specificity [7]. For next-generation sequencing, each sequencing experiment generates a large amount of sequence data, the analysis of which is a major bioinformatics challenge [8]. The substantial experimental costs, time-intensive sample pre-treatment, and complex experimental procedures associated with traditional miRNA detection methods significantly hinder their practical applicability [9,10,11]. Hence, it is crucial to devise a practical, rapid, sensitive, and cost-effective technique for detecting miRNA-122.

In the last few years, nucleic acid biosensors (NABs) have received significant attention for bioanalysis and clinical diagnosis because of their distinct advantages, including a high sensitivity and short assay time. NABs including electrochemical and optical NABs have been applied to detect miRNAs [12]. However, electrochemical NABs require specialized operators and it takes long time to prepare the electrodes [13,14,15]. Optical NABs require expensive instruments and strict reaction conditions [16,17]. The lateral flow nucleic acid biosensor (LFNAB), as a simple and rapid detection platform, overcomes the drawbacks of traditional NABs and offer some advantages, including simple operation, no need for complex instrumentation, instant visibility of the results, and a low cost. Recently, LFNABs have been used to detect miR-155 [18,19], miR-21 [20,21,22] and miR-210 [22,23] in cell lyses and serum samples. In this paper, an LFNAB is developed for the detection of miR-122. The optimized device exhibited highly linear responses within the range of 0 pM to 100 pM miR-122, with a detection limit of 3.90 pM. The LFNAB was utilized for detecting miR-122 in serum and fingertip blood, yielding satisfactory results.

## 2. Materials and Methods

### 2.1. Instruments

The XYZ Large Platform 3D Film Spraying Instrument, Microcomputer Automatic Chopper, CNC Strip Cutter, and Colloidal Gold Analyzer were produced by Shanghai Gold Standard Biotechnology Co., Ltd. (Shanghai, China), the Nano Particle Size and Zeta Potential Analyzer was bought from Dandong Baxter Instrument Co., Ltd. (Dandong, China), the high temperature oven was purchased from Shanghai Qixin Scientific Instrument Co., Ltd. (Shanghai, China), and the UV spectrophotometer was bought from Shanghai Yuan Analytical Instrument Co. (Shanghai, China).

### 2.2. Reagents and Materials

HAuCl_4_-3H_2_O (99.9%) was produced by Sigma-Aldrich, Inc. (St. Louis, MO, USA). N-butanol, Tween-20, polyethylene glycol, trisodium citrate (Na_3_Ct), and Triton X-100 were produced by Shanghai McLean Biochemistry Technology Co. (Shanghai, China). Glucose and sucrose were purchased from Sinopharm Chemical Reagent Co. (Shanghai, China). The 5× TBE buffer, Bovine serum albumin (BSA), 20× SSC buffer (pH = 7.0), and 10× PBS buffer (pH = 7.4) were bought from Solarbio. Absorbent paper was bought from Shanghai Gold Standard Biotechnology Co., Ltd. (Shanghai, China). Glass fibers and nitrocellulose membranes were bought from Shanghai Jiening Biotechnology Co., Ltd. (Shanghai, China).

Table 1 lists the oligonucleotides involved in this experiment, all of which were produced by Sangon Biotech. Co, Ltd. (Shanghai, China).

### 2.3. Preparation of Gold Nanoparticles (AuNPs)

AuNPs (average particle size of 28 nm) were prepared following previously reported methods with minor modifications [24]. First, 200 mL of 0.04% HAuCl_4_ was placed in a conical flask, the liquid level in the container was marked, the flask was put on a magnetic stirrer, and the sample was heated and stirred until boiling. To this solution, 4.7 mL of 2% trisodium citrate solution was added, accompanied by continuous heating and stirring. The color then changed from yellow to gray-black and finally to translucent red; then, heating was stopped and the solution was stirred at an appropriately low speed for a few minutes and cooled. After cooling to room temperature, the solution was replenished with water to the mark and kept away from light for use.

### 2.4. Preparation of Det-DNA-AuNP Conjugates

Det-DNA-AuNP conjugates were prepared in a manner similar to previously reported methods, with slight adjustments [25]. The procedure involved taking 50 µL of a 6-fold-concentrated AuNP solution, to which 2 µL of a 100 µmol/L Det-DNA probe was added and thoroughly mixed. The mixture was transferred into 1 mL of n-butanol and thoroughly mixed, and then 100 µL of 0.5× TBE was added and mixed well. The mixture was centrifuged at 2000 rpm for 20 s and the supernatant was discarded. The tiny black dot at the base of the centrifuge tube was preserved and then dispersed in 100 µL of distilled water (ddH_2_O). The above solution was centrifuged at 6000 rpm for 8 min. The liquid supernatant was removed and 100 µL of 1× PBS was added; the mixture was stirred at 6000 rpm and centrifuged for 8 min to remove any residual impurities. Finally, the resulting Det-DNA-AuNP conjugate was dispersed in 50 µL of resuspension buffer.

### 2.5. Preparation of LFNAB

The LFNAB included four elements: a sample pad, a conjugate pad, a nitrocellulose membrane, and an absorbent pad. All elements were placed on a PVC base plate. Among these components, the sample pad and conjugate pad were constructed from glass fiber. The sample pad was saturated with a solution comprising 0.5% Triton X-100, 2% glucose, 1% BSA, and 2% polyethylene glycol for a duration of 2 h. Similarly, the conjugate pad was immersed in 1× PBS containing 2% glucose, 1% BSA, 0.5% Triton X-100, and 2% polyethylene glycol, followed by drying at 37 °C. The major components including Triton X-100, glucose, BSA, and polyethylene glycol in the buffers play important roles in the preparation of the sample and conjugate pads. The addition of Triton X-100 to the buffer clears the surface of the fibers of oil, enhances wettability, and allows the subsequent components to adhere evenly. Glucose regulates osmotic pressure, maintains the stability of the physical structure of the glass fibers, and prevents the growth of microorganisms. BSA modifies the surface to reduce the non-specific adsorption of biological samples and stabilize the protein system. The addition of polyethylene glycol prevents fiber adhesion and adjusts the viscosity of the solution to help with uniform processing. The absorbent pad material was absorbent paper. Both the glass fiber and absorbent paper were cut into long strips of different widths using a microcomputerized automatic chopper, and then formed into sample pads, conjugate pads, and absorbent pads. The Det-DNA/AuNP coupling solution was sprayed onto the binding mat with a XYZ large-platform 3D film-sprayer and dried at 37 °C for 2 h. To immobilize the biotinylated Cap-DNA and Con-DNA on the nitrocellulose membrane, streptavidin was used to bind the biotinylated Cap-DNA and Con-DNA, creating streptavidin-biotinylated Cap-DNA and streptavidin-biotinylated Con-DNA complexes. These complexes were then applied onto the nitrocellulose membrane using an XYZ large-platform 3D film sprayer to create the test and control areas. The nitrocellulose membrane was dried at 37 °C for 2 h. Subsequently, the sample pads, conjugate pads, nitrocellulose membranes, and absorbent pads were collected and cut using a CNC cutting machine to create the test strips.

### 2.6. Sample Analysis Procedure

For miR-122 standard testing, a range of sample solutions with different miR-122 concentrations were created using 4× SSC as the up-sampling buffer, with 75 µL utilized for each test. After applying the sample solution on the LFNAB and waiting for 15 min, the test and control areas could be visually examined. Qualitative analysis was performed by simply observing the red bands located in the test and control areas. In addition, two red bands appeared on both the test area and control area indicating a positive (+) test; only one red band on the control area indicates a negative (−) test and no band on either the test area or control area, or a red band appearing only on the test area, indicates that the test is invalid. Quantitative analysis was performed using a colloidal gold analyzer to read the color intensity data for the test and control lines. Finally, calibration curves were plotted using the ratios of the test line intensity to the control line intensity against miRNA-122 concentrations.

For the real sample test, serum, fingertip blood, spiked serum, and spiked fingertip blood were used as samples. First, we tested normal human serum and serum spiked with miR-122, plotted the standard curve and calculated its recovery. Normal human serum was diluted 10 times with 4× SSC. The spiked serum samples were prepared by spiking the diluted serum with miR-122. For the strip test, 75 µL of the diluted serum (or the spiked serum) was used. Before adding the blood sample to the test strip, we diluted the blood sample with 4× SSC buffer, mixing 10 μL of healthy human blood sample with 65 μL of 4× SSC buffer, and then used a pipette to take 75 μL of the mixed solution and load it onto the test strip for detection.

## 3. Results

### 3.1. The Principle of Testing miR-122 on LFNAB

**Figure 1:**
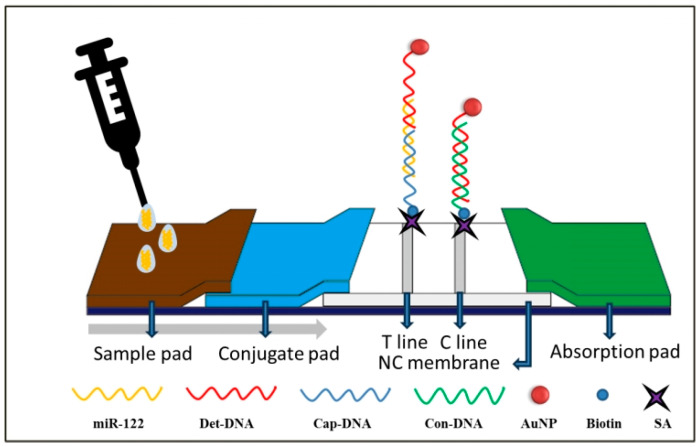
Schematic diagram of LFNAB for the miR-122 assay.

The principle of detecting miR-122 on LFNAB (Figure 1) relies on a dual DNA-RNA hybridization reaction. After the sample solution containing miR-122 is added to the sample pad, the solution will flow along the LFNAB due to capillary action. And when it flows through the conjugate pad, the miR-122 will undergo DNA-RNA hybridization with AuNP/Det-DNA on the conjugate pad, generating the AuNP/Det-DNA-miR-122 complexes, and when flowing through the test area, the AuNP/Det-DNA-miR-122 complexes will be captured by the second DNA-RNA hybridization reaction between the miR-122 and the Cap-DNAs on the test line. This interaction will lead to the formation of AuNP/Det-DNA-miR-122-Cap-DNA complexes, where AuNPs are enriched and show a red band, i.e., the T line. Excess AuNP-Det-DNA conjugate flows through the control region and hybridizes with Con-DNA on the control line, generating the AuNP/Det-DNA-Con-DNA complexes, where AuNPs are enriched, showing the second red band, the C line. When the sample solution does not contain miR-122, only the C line is shown. The total time taken to complete the assay is 15 min. For qualitative detection (Yes/No), one can observe the T line and C line on the tested strips. If both the T line and C line are shown on the tested strip, it indicates the sample solution contains miR-122 (positive); if only C line is shown on the tested strip, it indicates that the sample solution does not contain miR-122 (negative), but if only the T line is shown on the tested strip or no line is shown on both T line and C line, it indicates that the test is invalid. It is noted that the intensity of the T line increases with increasing miR-122 concentration. For quantitative detection, a colloidal gold analyzer is used to determine the concentration of miR-122. The tested strips are inserted into the colloidal gold analyzer for imaging and to determine the gray values of the T and C lines. Thus, the ratios of T/C are used to plot the calibration curve. In order to ensure that the nanoparticle deposition is homogeneous, first, we prepared relatively homogeneous gold nanoparticles (as shown in Figure S1 in the supporting literature); second, we used the gold spraying and scribing instrument, using a scribing speed of 1 μL/cm and a gold spraying speed of 6 μL/cm, and selected the part with the most uniform scribing and gold spraying after drying.

### 3.2. Optimization of Experimental Parameters

The experimental conditions have a great influence on the sensitivity of the analysis of miR-122 on the LFNAB. We optimized several experimental conditions including the sources of nitrocellulose membranes (a), the molar ratio of streptavidin (SA) to the biotinylated DNA probe (b), the concentration of AuNPs used to prepare the Det-DNA-AuNP conjugate (c), the amount of Det-DNA probe (d), the amount of AuNP-Det-DNA conjugate per strip (e), and the concentration of running buffer (f). Figure S2 in the ESI provides the detailed experimental conditions and further discussion of them. The optimized conditions were (a) a nitrocellulose membrane using CN140; (b) a 3:1 ratio of biotin-modified probe immobilized on the TC line to streptavidin; (c) a 6-fold concentration of AuNP solution; (d) 2 µL of Det-DNA probe; (e) 1.8 µL of conjugate per strip; and (f) 4x SSC as the running buffer.

### 3.3. Analytical Properties

**Figure 2:**
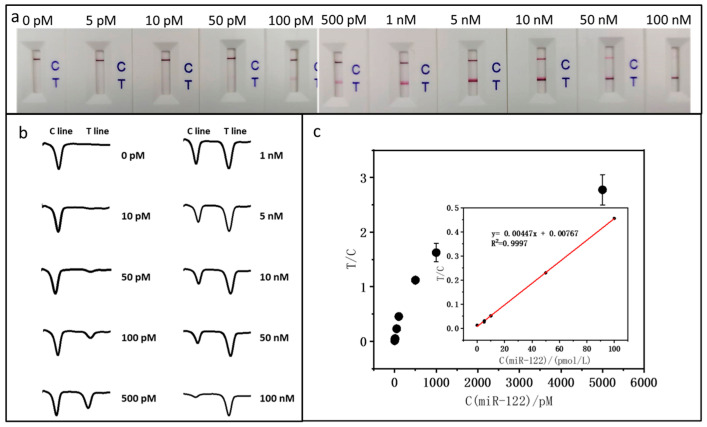
(**a**) Photographs of LFNABs after testing various miR-122 concentrations; (**b**) corresponding responses of LFNABs obtained from colloidal gold analyzer; (**c**) calibration curve for miR-122 show in (**b**). Inset: the linear range region.

Sample solutions with varying miR-122 concentrations were tested under the optimal conditions to evaluate the LFNAB’s analytical performance. To minimize the error, six sets at each concentration level were prepared and each set of tests was repeated three times. Quantitative analysis was performed with a colloidal gold analyzer, and the average of three repetitions of the tests was taken as a single set of data, and the average of the six sets was taken to plot the calibration curve. Figure 2a presents photographic images of the LFNABs following tests with varying concentrations of miR-122. It can be observed that the intensity of the T line increases as the concentration of miR-122 rises, and the intensity of the C line maintains a constant level within the concentration range from 0 to 1 nM, then decreases at higher concentrations. The reduction in C line intensity is attributed to the consistent quantity of AuNP-Det-DNA dispensed on the conjugate pad of the LFNAB. With a greater number of AuNP-Det-DNA conjugates captured on the T line and a diminished number captured on the C line, the T/C ratio is consequently leveraged to establish the calibration curve. It is noted that there is no T line in the absence of miR-122 (control), indicating that there is no nonspecific adsorption on the test area under the optimal experimental conditions. The lowest detectable miR-122 concentration is 10 pM, which is used was the limit of qualitative detection. Quantitative data were obtained by reading the gray values (intensities) of the T and C lines with the colloidal gold analyzer. Figure 2b shows the corresponding responses of the LFNABs tested in Figure 2a. When miR-122 is present, there are two peaks, while in the absence of miR-122, there is only one peak. The peak area presents the intensities of the T and C lines. Figure 2c shows the calibration curve for miR-122 detection, and the interpolation plot shows good linear dynamics in the 0 pM to 100 pM range. According to the standard deviation (SD) of 3, the quantitative detection limit is estimated to be around 3.90 pM.

### 3.4. Specificity Test

**Figure 3:**
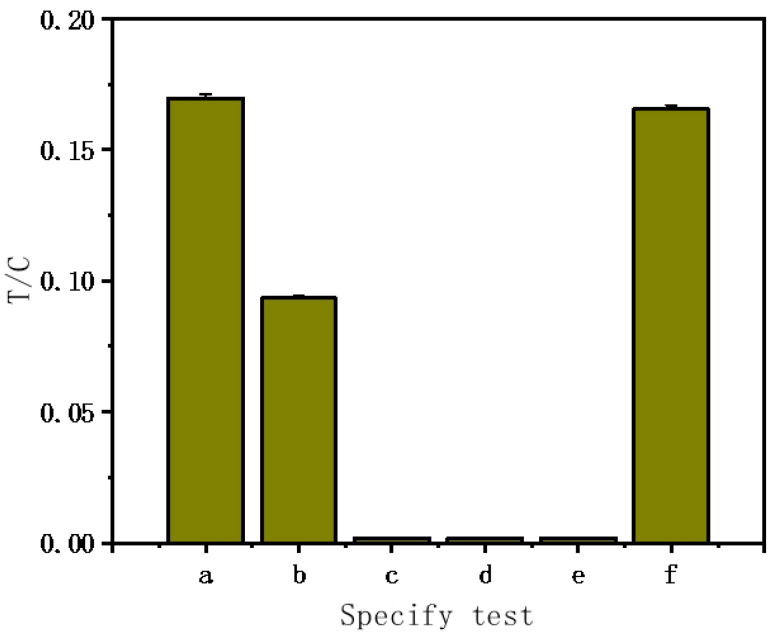
MiR-122 specificity tests: (a) 50 pM miR-122; (b) 50 pM single-base mismatched miRNA; (c) 50 pM double-base mismatched miRNA; (d) 50 pM triple-base mismatched miRNA; (e) 500 pM random miRNA; (f) 50 pM miR-122 + 500 pM random miRNA.

To assess the LFNAB’s specificity for miR-122 detection, we set up six groups of experiments, namely (a) 50 pM miR-122; (b) 50 pM single-base mismatched miRNA; (c) 50 pM two-base mismatched miRNA; (d) 50 pM three-base mismatched miRNA; (e) 500 pM random miRNA; (f) 50 pM miR-122 + 500 pM random miRNA. As can be seen, negligible responses were obtained with two-base mismatch, three-base mismatch, and non-complementary miRNA; the highest response was obtained with the target miR-122 (Figure 3). It is worth noting that the LFNAB can detect a single mismatch in the target miR-122, the response of one-mismatch microRNA is around 50% of the response of miR-122. Furthermore, the responses of miR-122 and the random miRNA mixture are similar to that of miR-122, indicating that the random miRNA has no effect on the detection of miR-122.

### 3.5. Detection of miR-122 in Serum and Finger Blood

#### 3.5.1. Detection of miR-122 in Serum

**Figure 4:**
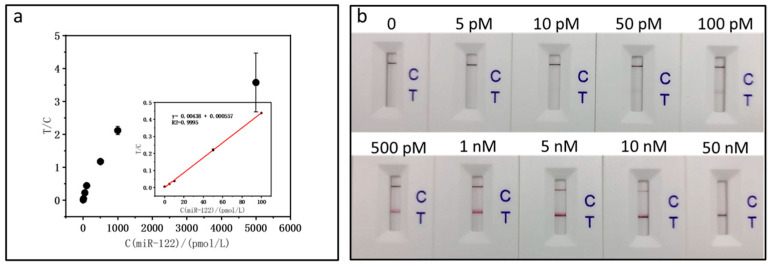
Calibration curves (**a**) and photographs (**b**) of test strip assays for detecting varying concentrations of miR-122 in serum.

As shown in Figure 4, after spiking the normal human serum, we plotted the calibration curve with the equation T/C = 0.00438 C(miR-122)/(pmol/L) + 0.000557, and the correlation coefficient was 0.9995, which is a little less linear than that of miR-122 without spiking the serum. The sensitivity of the miR-122 test for the spiked serum sample matrix was less than that for the pure buffer. Based on the test strip detection photos, it can be seen that the sensitivity of miR-122 in serum is higher than that in the standard solutions at a concentration of 10 pM. According to the standard deviation (SD) of 3, the quantitative detection limit is estimated to be around 4.09 pM. Moreover, we calculated the recovery of miR-122 in serum (Table 2), which was good.

#### 3.5.2. Detecting miR-122 in Fingertip Blood

**Figure 5:**
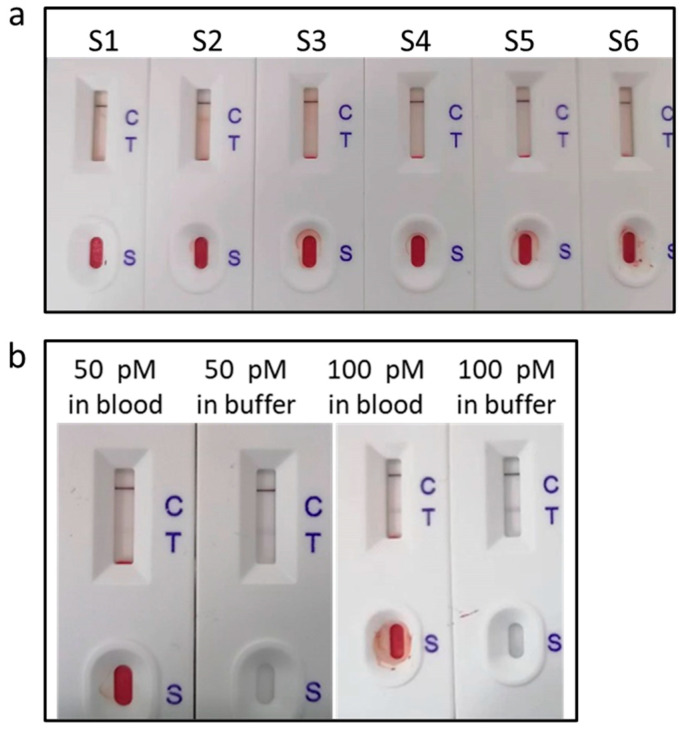
(**a**) Photographs of LFNABS after testing fingertip blood samples from six healthy individuals; (**b**) photographs of LFNABS after testing fingertip blood sample spiked 50 pM and 100 pM miRNA-122.

We tested fingertip blood samples from six healthy individuals. Figure 5a shows photographs of the LFNABs. One can see only the C line and no T line, indicating that the concentration of miRNA-122 is below 10 pM. Afterwards, the fingertip blood samples were spiked with 50 pM and 100 pM miRNA-122 and tested (Figure 5b), which showed the T line, indicating that fingertip blood hardly interferes with miR-122 detection.

## 4. Discussion

In conclusion, this study successfully demonstrated the detection of microRNA-122 using a lateral flow biosensor with a visual detection limit of 10 pM. By comparison, Gao et al. [26] reported a DNA-gold nanoparticle (DNA-GNP)-based lateral flow nucleic acid biosensor that, after systematic optimization, achieved a detection limit of 60 pM for miRNA-215. Our sensor demonstrated a six-fold improvement in sensitivity, which can be primarily attributed to the use of butanol dehydration in the preparation of the conjugate. Ding et al. [27] systematically compared five methods—salt aging, microwave-assisted dry heating, freezing and thawing, low pH treatment, and butanol dehydration—evaluating their effects on the analytical performance of lateral flow nucleic acid biosensors (LFNABs). The findings confirmed that the conjugates prepared using butanol dehydration achieved the lowest detection limits, further validating the superiority of this method.

Despite these advances in sensitivity, the application of lateral flow sensors for whole-blood testing remains challenging. In order to demonstrate the potential applications of this biosensor, we tested the concentrations of miR-122 in the clinical samples including healthy and patient serum and finger blood, but there no response was obtained due to the high detection limit of the lateral flow nucleic acid biosensor (3.9 pM). The concentration of miRNA-122 in healthy and patient serum is below 1 pM. In order to demonstrate the potential applications of the biosensor, we used spiked serum samples in the tests of specificity and sensitivity for comparison. Specifically, in serum and fingertip blood testing, the need for the addition of a buffer to dilute samples results in reduced microRNA concentrations, thereby limiting their practical applicability. Table S1 in ESI summarizes the existing miR-122 detection methods, with a focus on comparing detection limit, reaction time, and other relevant parameters. The detection limit of the LFNAB (3.9 pM) in this research is comparable with some of the existing miR-122 assays (Microarray, fluorescent bioassay, electrochemical detection); however, the sensitivity of LFNAB is not enough to detect miR-122 directly in clinical samples. Consequently, developing methods capable of achieving highly sensitive detection directly in whole blood and serum has become a critical area of research.

To address this limitation, several advanced isothermal nucleic acid amplification technologies have shown significant potential. Recombinase polymerase amplification (RPA) is a rapid and highly sensitive isothermal amplification technique that facilitates efficient target nucleic acid amplification at low temperatures [28]. Loop-mediated isothermal amplification (LAMP) is recognized for its high specificity and rapid amplification, making it particularly suitable for detecting stable targets [29]. CRISPR systems, especially those utilizing Cas12 and Cas13 proteins, offer exceptional potential for the sensitive detection of short nucleic acids like microRNAs due to their highly specific target recognition and cleavage properties [30]. Similarly, the exponential amplification reaction (EXPAR) is a highly efficient isothermal amplification technique that rapidly amplifies short nucleic acid sequences, making it especially effective for detecting microRNAs and other small-molecule targets [31].

In this context, integrating lateral flow sensors with advanced techniques such as RPA, LAMP, CRISPR, and EXPAR represents a pivotal strategy to enhance microRNA detection performance. Among these, EXPAR stands out for its comparable amplification efficiency to other isothermal techniques, such as rolling circle amplification (RCA), LAMP, and RPA, while eliminating the need for thermal cycling [32]. This characteristic significantly improves its suitability for both point-of-care and real-time applications. Consequently, combining lateral flow assays (LFAs) with EXPAR to enable the direct and rapid detection of microRNAs is anticipated to become a key focus leading to breakthroughs in future research.

## 5. Conclusions

In this paper, we employed a lateral flow biosensor to detect microRNA-122 in human serum, and after system optimization, we could detect miR-122 at 4.09 pM. The successful utilization of the biosensor for detecting microRNA in spiked fingertip blood, with the potential to characterize the degree of liver injury in humans, marks a significant advancement. The next step is to improve the detection sensitivity and apply it in clinical practice for the detection of microRNA in patients with different stages of hepatocellular carcinoma, to achieve early diagnosis, early detection, and early treatment.

## Figures and Tables

**Table 1:** The oligonucleotide sequences used in this experiment.

Name	Sequence (5′–3′)
miR-122	UGGAGUGUGACAAUGGUGUUUG
Thiolated detection DNA probe (Det-DNA)	thiol-CCCCCCAAACACCATT
Biotinylated capture DNA probe (Cap-DNA)	GTCACACTCCACCCCC/Biotin
Biotinylated control DNA probe (Con-DNA)	Biotin/AATGGTGTTTGGGGGG
random miRNA	UUGUACUACACAAAAGUACUG
Single-base mismatched miRNA	UGGAGCGUGACAAUGGUGUUUG
Double-base mis-matched miRNA	UGGAGCGUGACAAUAGUGUUUG
Triple-base mismatched miRNA	UGGAGCGUGACAAUAGUGUUCG

**Table 2:** Recovery of miR-122 in serum.

Serum Sample	Added (pM)	Found (pM)	Recovery (%)	RSD (1/4^3^)
1	5	4.76	95.2	0.98
2	50	50.79	101.6	0.05
3	100	99.83	99.8	0.08

## Data Availability

The original contributions presented in this study are included in the article/Supplementary Materials. Further inquiries can be directed to the corresponding authors.

## Supplementary Materials

The following supporting information can be downloaded at: https://www.mdpi.com/article/10.3390/bios15010058/s1. The Figure S1. Characterization of AuNPs and AuNP-Det-DNA conjugates, Figure S2. Plot of optimized experimental conditions and Table S1. Comparison of different detection methods for miR-122 [33,34,35,36,37,38,39,40,41,42,43].
