# Supplementary material for: Rapid Detection of microRNA-122 in Serum and Finger Blood Using a Lateral Flow Nucleic Acid Biosensor
Source: Biosensors (Basel). 2025 Jan 17;15(1):58. doi: 10.3390/bios15010058 (PMC11764104; doi:10.3390/bios15010058)
Supplement: Supplementary file 1 [file biosensors-15-00058-s001.zip › biosensors-3379169-supplementary.pdf]

Electronic Support Information

Rapid detection of microRNA-122 in serum and finger blood using a lateral flow nucleic acid biosensor

Min Zhang [a], Meijing Ma [a], Jiahui Wang [a], Yurui Zhou [a], Xueji Zhang [b]† and Guodong Liu [a]†

Characterization of AuNPs and AuNP-Det-DNA conjugates

Figure S1(a) shows a typical TEM image of the as-prepared AuNPs. It can be seen that the diameter of the AuNPs was around 28 nm; the distribution of nanoparticle size was uniform (Figure S1(b)). Figure S1(c) presents the UV absorption spectrum of the AuNP (black curve) and the AuNP-Det-DNA conjugate (red curve). A typical UV absorption peak at 525 nm was observed with the AuNP solution, and a new UV absorption peak at 260 nm was obtained with the AuNP-Det-DNA conjugate solution. The new absorption peak at 260 nm is a typical DNA absorption peak, indicating the Det-DNA probes were immobilized on the AuNP's surface successfully.

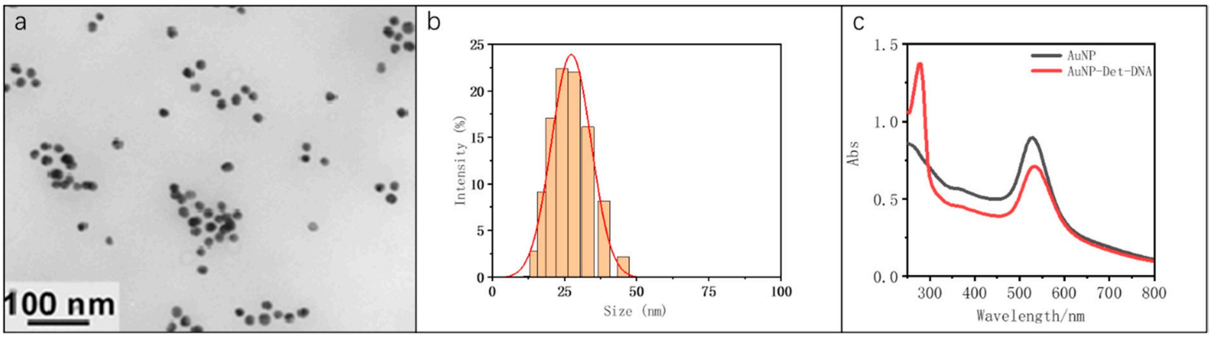

Figure S1. Characterization of AuNPs and AuNP-Det-DNA conjugates. (a) TEM image of AuNPs; (b) statistical plot of AuNP size distribution; (c) UV absorption curves of AuNPs before and after conjugating with Det-DNA probes.

Optimization of experimental parameters

To optimize the conditions, we used two detection concentrations, 0 and 25 pM, and experimented with three parallel groups, and finally took their averages.

Optimization of the types of nitrocellulose membranes: We tried three different nitrocellulose membranes, which were CN140, VIVID, and JN140. As can be seen from **Figure S2(a)**, the test strip prepared with CN140 film has the largest signal-to-noise ratio and the best effect when used for detection.

Optimization of the molar ratio between streptavidin and biotinylated Det-DNA: Theoretically, 1 mol of streptavidin can be combined with 4 mol of biotin. Therefore, we optimized this experiment with different ratios of these two substances. As can be seen in **Figure S2(b)**, when the molar ratio of streptavidin to biotinylated Det-DNA is 1:3, the test strip has the best detection effect.

Optimization of the concentration ratio of AuNPs: Increasing the concentration of gold nanoparticles can improve the detection sensitivity, so we concentrated the gold nanoparticle solution by different folds. We concentrated the initial concentrations of the gold nanoparticle solution 4, 6, 8, and 10 fold. During the detection process, it was found that, as the concentration of gold nanoparticles increased, the detected concentration became lower and lower, but when the concentration of gold nanoparticles increased to a certain range, our blank test strip would show a T-line, that is, there was a background signal. As shown in **Figure S2(c)**, the optimal concentration factor for gold nanoparticles was found to be 6 fold.

Optimization of the amount of Det-DNA: We mixed the detection probe with gold nanoparticles, and used the butanol dehydration reaction to form Au-S bonds, which in turn achieved the goal of color development. Generally speaking, when a gold nanoparticle is connected to a Det-DNA, the sensitivity is the highest, which means that there are as many gold nanoparticles as there are Det-DNA, and the more gold nanoparticles and the darker the color, the stronger the signal. If there is an excessive amount of detection probes, one gold nanoparticle will be linked to multiple Det-DNA probes, and fewer gold nanoparticles will accumulate and the signal will be weaker. As shown in **Figure S2(d)**, the signal is strongest when 2 μL of Det-DNA is added.

Optimization of the amount of conjugates per strip: As mentioned above, the more gold nanoparticles, the darker the color and the stronger the signal. In general, the larger the volume of the conjugate, the stronger the signal, but an excess of the conjugate can cause non-specific adsorption, resulting in background signals and a decrease in S/N values. Our gold spraying instrument was set to x μL/cm, the width of each strip was 3mm, and the amount of conjugate was a multiple of 3; therefore, we optimized the values to 1.5 μL, 1.8 μL, 2.1 μL, 2.4 μL. As shown in **Figure S2(e)**, the optimal amount was 6 μL/cm, i.e., 1.8 μL.

Optimization of the concentration of SSC buffer: Saline solution can promote the hybridization of DNA molecules. SSC buffer is the standard blotting and hybridization solution in molecular biology. The sodium citrate in the SSC buffer acts as a buffer, and the salt ions neutralize the negative charge on the nucleic acid backbone, making it electrically neutral, which makes it easier to bind the probe to the target sequence. The usual concentration is 2 x. As shown in **Figure S2(f)**, we optimized the original concentration of 20 x SSC buffer, with a 4 x SSC buffer showing the optimal results.

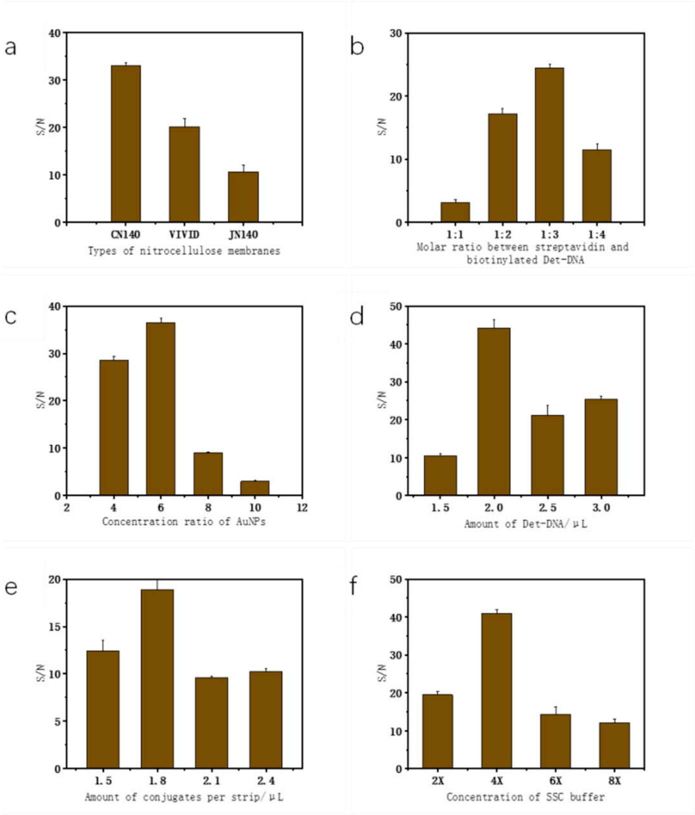

Figure S2. Plot of optimized experimental conditions. (a) Types of cellulose nitrate membranes; (b) molar ratio between streptavidin and biotinylated Det-DNA; (c) concentration ratio of AuNPs; (d) Amount of Det-DNA; (e) amount of conjugates per strip; (f) concentration of SSC buffer.

Comparison of different detection methods for miR-122

As shown in Table S1, most of the miR-122 detection methods are time-consuming, costly, and require large instruments, and the lateral flow nucleic acid biosensor used in this paper only takes 15-30 min from preparation to the detection of miR-122, which is fast and low-cost, and the results can be visually detected without the need for large instruments.

Table S1. Comparison of different detection methods for miR-122.

Method	Sample type	Detection limit	Detection time	Whether an instrument is required	Ref.
RT-qPCR	rat liver	a few thousand molecules	45 min	Real-time PCR instrument	[33]
Single nucleobase labelling	human serum	0.3 nM	-	MAGPIX instruments	[34]
Single molecule arrays	human serum	500 fM	3.5 h	Simoa HD-1 Analyzer, Fluorescent imager with sub-micron resolution	[35]
Microarray	spiked serum	0.043 nM	-	Scan-express instrument	[36]
In situ synthesis	human serum	4.1 fM	-	Potentiostats	[37]
Electrochemical detection	tissues	50 pM	-	Potentiostats	[38]
SERS	/	7.75 aM	-	Raman spectroscopy	[39]
Fluorescence bioassay	spiked serum	84 pM	within 45 min	LS-55 luminescence spectrometer	[40]
Laser-induced fluorescence (LIF) detection	chicken and duck livers	0.60 pM	45 min	Confocal LIF detector	[41]
Biomimetic nanochannel logic platform	human liver	97.2 aM	2.5 h	Keithley 6487 picoammeter	[42]
Extended-based dual-driven DNA molecular machine (E-DDM)	serum	0.82 fM	1.5 h	Fluorescence spectrophotometer	[43]
Lateral flow nucleic acid biosensor	spiked serum	3.90 pM	30 min	Colloidal Gold Analyzer	This paper

Reference

33. Jin, J., Vaud, S., Zhelkovsky, A. M., Posfai, J., and McReynolds, L. A., Sensitive and specific miRNA detection method using SplintR Ligase, Nucleic Acids Res 44(13) (2016) e116.
34. Venkateswaran, S., Luque-González, M. A., Tabraue-Chávez, M., Fara, M. A., López-Longarela, B., Cano-Cortes, V., López-Delgado, F. J., Sánchez-Martín, R. M., Ilyine, H., and Bradley, M., Novel bead-based platform for direct detection of unlabelled nucleic acids through Single Nucleobase Labelling, Talanta 161 (2016) 489-496.
35. Rissin, D. M., Lopez-Longarela, B., Pernagallo, S., Ilyine, H., Vliegenthart, A. D. B., Dear, J. W., Diaz-Mochon, J. J., and Duffy, D. C., Polymerase-free measurement of microRNA-122 with single base specificity using single molecule arrays: Detection of drug-induced liver injury, PLoS One 12(7) (2017) e0179669.
36. Forte, G., Ventimiglia, G., Pesaturo, M., and Petralia, S., A highly sensitive PNA-microarray system for miRNA122 recognition, Bi-otechnol J 17(6) (2022) e2100587.
37. Shen, H., Li, Z., Dou, B., Feng, Q., and Wang, P., An amplified logic gate driven by in situ synthesis of silver nanoclusters for iden-tification of biomarkers, Chem Commun (Camb) 59(38) (2023) 5705-5708.
38. Roychoudhury, A., Dear, J. W., Kersaudy-Kerhoas, M., and Bachmann, T. T., Amplification-free electrochemical biosensor detection of circulating microRNA to identify drug-induced liver injury, Biosens Bioelectron 231 (2023) 115298.

39. He, Y., Yang, X., Yuan, R., and Chai, Y., A novel ratiometric SERS biosensor with one Raman probe for ultrasensitive microRNA detection based on DNA hydrogel amplification, J Mater Chem B 7(16) (2019) 2643-2647.
40. Tan, L., Fu, S., Lu, J., Hu, K., Liang, X., Li, Q., Zhao, S., and Tian, J., Detection of microRNA using enzyme-assisted amplifying and DNA-templated silver nanoclusters signal-off fluorescence bioassay, Talanta 210 (2020) 120623.
41. Yu, X., Zhang, S., and Wang, W., Determination of microRNA-122 in hepatocytes by two-step amplification of duplex-specific nu-clease with laser-induced fluorescence detection, Anal Methods 14(17) (2022) 1715-1720.
42. Zhang, S., Cheng, J., Shi, W., Li, K. B., Han, D. M., and Xu, J. J., Fabrication of a Biomimetic Nanochannel Logic Platform and Its Ap-plications in the Intelligent Detection of miRNA Related to Liver Cancer, Anal Chem 92(8) (2020) 5952-5959.
43. Zhang, C., Zhu, F., Chen, Y., He, L., Zhang, T., Zhou, B., Ge, C., Wang, J., and Wu, B., Breaking barriers: Overcoming low abundance of miR-122 with E-DDM for precise detection in HCC patients, Sensors and Actuators B: Chemical 405 (2024) 135372.
